# A critique of the Uganda district league table using a normative health system performance assessment framework

**DOI:** 10.1186/s12913-018-3126-6

**Published:** 2018-05-10

**Authors:** Christine KirungaTashobya, Freddie Ssengooba, Juliet Nabyonga-Orem, Juliet Bataringaya, Jean Macq, Bruno Marchal, Timothy Musila, Bart Criel

**Affiliations:** 1 Health Policy and Planning Department, School of Public Health Makerere University, New Mulago Hill, P.O Box 7072, Kampala, Uganda; 2 World Health Organization, Inter-Country Support Team for Eastern and Southern Africa; Health Systems and Services Cluster, P.O Box CY 348: Causeway, Harare, Zimbabwe; 3 Health Systems and Services Cluster, World Health Organization Rwanda Country Office, Boite Postale, 1324 Kigali, Rwanda; 4 Institute of Health and Society, Catholic University of Louvain, Promenade de l’Alma, B-12000 Brussels, Belgium; 5 Public Health Department, Institute of Tropical Medicine, 155 Nationalestraat, 2000 Antwerp, Belgium; 6 Ministry of Health, 6 Lourdel Road, Nakasero, P.O Box 7272, Kampala, Uganda

**Keywords:** District, Health system, Performance assessment, Accountability, Decision-making, League table, Decentralization

## Abstract

**Background:**

In 2003 the Uganda Ministry of Health (MoH) introduced the District League Table (DLT) to track district performance. This review of the DLT is intended to add to the evidence base on Health Systems Performance Assessment (HSPA) globally, with emphasis on Low and Middle Income Countries (LMICs), and provide recommendations for adjustments to the current Ugandan reality.

**Methods:**

A normative HSPA framework was used to inform the development of a Key Informant Interview (KII) tool. Thirty Key Informants were interviewed, purposively selected from the Ugandan health system on the basis of having developed or used the DLT. KII data and information from published and grey literature on the Uganda health system was analyzed using deductive analysis.

**Results:**

Stakeholder involvement in the development of the DLT was limited, including MoH officials and development partners, and a few district technical managers. Uganda policy documents articulate a conceptually broad health system whereas the DLT focuses on a healthcare system. The complexity and dynamism of the Uganda health system was insufficiently acknowledged by the HSPA framework. Though DLT objectives and indicators were articulated, there was no conceptual reference model and lack of clarity on the constitutive dimensions. The DLT mechanisms for change were not explicit. The DLT compared markedly different districts and did not identify factors behind observed performance. Uganda lacks a designated institutional unit for the analysis and presentation of HSPA data, and there are challenges in data quality and range.

**Conclusions:**

The critique of the DLT using a normative model supported the development of recommendation for Uganda district HSPA and provides lessons for other LMICs. A similar approach can be used by researchers and policy makers elsewhere for the review and development of other frameworks.

Adjustments in Uganda district HSPA should consider: wider stakeholder involvement with more district managers including political, administrative and technical; better anchoring within the national health system framework; integration of the notion of complexity in the design of the framework; and emphasis on facilitating district decision-making and learning. There is need to improve data quality and range and additional approaches for data analysis and presentation.

## Background

Efforts in assessing performance of health systems can be traced back almost three centuries, although most of the theoretical and empirical work in health system performance assessment (HSPA) has taken place in the last three decades [1–3]. One of the approaches that has been used for HSPA is the league table [4, 5]. The ultimate purpose of HSPA is to improve the quality of decisions by stakeholders in the health system, thereby contributing to health system improvements. The design, process of development and implementation of the HSPA frameworks should facilitate the achievement of this purpose [6, 7].

Uganda is a low income country (LIC) in sub-Sahara Africa with a Gross National Income (GNI) per capita of current US $ 670 (2014) and a high burden of disease (both communicable and non-communicable) [8]. Although some improvements have been registered over the last three decades, the country still has poor health indices with infant mortality rate at 43 deaths per 1000 live births (2016) and maternal mortality ratio at 336 deaths per 100,000 live births (2016) [9]. Total health expenditure at US $ 53 per capita is very low; recent estimates indicate the following mix: public 15.3%; private 38.4% and development partners 46.3% [10]. The model of governance practiced in the country is the devolution form of decentralization, with political, administrative and technical authority at the national, district and sub-county levels [11]. The central level is responsible for legislation, policy formulation and strategic planning, resource mobilization and monitoring and evaluation. The district is responsible for operational planning and management of health services, and carries the responsibility for inter-sectoral coordination of activities designed to improve population health [12].

In 2003 the Ministry of Health (MoH) introduced the Uganda District League Table (DLT) to track district performance given decentralized service delivery and the need to know the range of performance across the country [13]. The objective of this study was to carry out a comprehensive critique of the Uganda DLT using a normative HSPA framework. The review was intended to provide recommendations for improving Uganda’s district HSPA, and to provide lessons to other low and middle income countries (LMICs) with similar context like Uganda’s, as well as organizations seeking to develop or modify their HSPA frameworks.

### A model HSPA framework

Many of the HSPA experiences that have been studied have been developed in high income countries (HICs) [3, 14]. Although there are marked differences in contexts between HICs and LMICs, theoretical models and experiences of HSPA developed in one context can be used to inform the study and practice of HSPA in other contexts [14, 15]. A broad research programme on HSPA sought to learn from theoretical and empirical work on HSPA in different contexts to inform the development of new or review of existing HSPA frameworks in LMICs. The research programme was constituted by researchers based in Uganda, Belgium and the World Health Organization (WHO). The first author and four of the co-authors had been involved in the development and/or implementation of the DLT. The experience had stimulated an interest in learning about HSPA frameworks and what makes them appropriate (or not) for their purpose.

In the first stage of the research programme a model HSPA framework was developed for the purpose of reviewing HSPA frameworks for their appropriateness [16]. A structured literature review was carried out for the purpose of identifying characteristics of a ‘good’ HSPA framework. The review was initiated with a search of the PubMed database using the search term ‘health system performance assessment’. A total of 2522 articles published in English between 1995 and 2013 were identified. A review of titles, abstracts and eventually the full articles led to the identification of 16 relevant articles, 28 more articles were identified from the bibliography, making a total of 44 relevant articles [16]. A number of characteristics for a ‘good’ HSPA framework were extracted from the articles, which were summarized into 6 attributes by the researchers. The six attributes of a ‘good’ HSPA framework covered: the process of development; the relationship with the health systems framework; the relationship with the policy organizational and societal context; the elaboration of the framework; the institutional set up for HSPA; and the mechanisms for eliciting change in the health system. The attributes were presented to a group of Ugandan based experts for the purpose of providing broader input into the process, increasing objectivity, validating the findings and improving uptake of findings in the Ugandan decision-making processes. The individuals selected for the expert group were those with a minimum of postgraduate qualifications in public health/health economics/social sciences, and at least 10 years’ experience in health system management [16]. The expert group validated the six attribute model HSPA framework, provided some fresh perspectives, and introduced a seventh attribute covering the adaptability of a framework in different contexts and over time. The seven attributes are presented in Table 1. The resulting set of seven attributes was used to review a number of HSPA frameworks selected from high, middle and low income countries, with the objective of determining their responsiveness, and facilitating lesson learning for LMICs seeking to develop and/or review their HSPA frameworks. This process also served to determine the appropriateness of the model for critiquing HSPA frameworks [16]. The model for a HSPA framework thus developed and validated through these processes was utilized to review the DLT in this paper.

**Table 1:** Attributes of a normative HSPA Framework

***Process of development*** **(*and review*)** of the framework should be inclusive, with the participation of key stakeholders, and involve the explicit use of evidence to indicate causal links. *Embedded in an* ***explicit health system conceptual model***, including the determinants of health, system goals, constitutive elements, and actors. *Relate to the* ***national policy, organizational set-up and societal contex****t* including consideration of the level of development, epidemiological and demographic patterns, mode of government, levels and sources of health financing, governance, principles and values of society. *Well developed with* ***a conceptual model, a clear purpose, dimensions and sub-dimensions, and with appropriate indicators*** **.** Supported by an ***institutional set-up for performance assessment*** with appropriate resources and networks, including champions for performance assessment and an enabling environment. Explicitly *provide* ***mechanisms for eliciting change*** *in the health system* – indicating how the measurement of performance is linked to changes in policy, management, and delivery of services by various levels and players in the health system. ***Adaptable*** *to different contexts-* with history of use and or adaptation in different contexts, the length of time it has been in use and changes made to improve or adjust the framework in view of major reforms in the health system or elsewhere. ***Source: Tashobya*** **et al.** ***, 2014***	

## Methods

The study documented in this paper is a component of a broader research programme on the appropriateness of HSPA frameworks organized in three stages. The first stage focused on the development of a model HSPA framework as reported in the previous section [16]. The second stage of the research programme was a critique of a HSPA framework, which utilized the Uganda DLT as a case study. In the third stage of the research programme, the findings of the first and second stages will be used to inform the design of an adjusted district HSPA framework for Uganda, and to provide lessons for policy makers and researchers in other LMICs seeking to review or develop HSPA frameworks.

In the second stage of the programme, qualitative and quantitative research approaches were utilized to provide a comprehensive critique of the Uganda DLT. Qualitative data was sought from Key informant Interviews (KIIs) and grey and published literature. The model HSPA framework together with findings from literature, and the field knowledge of the Uganda-based researchers were used to develop an open-ended interview guide. Individuals to be interviewed were purposively selected from among health sector stakeholders given experience with the development, implementation, and/or use of information from the DLT. Interviewees were individuals working with the government at the national or local government levels, international agencies, researchers, and public and private sector players. The documents selected for review provided information on the Ugandan health system context over the last 20 years, and on the development and use of the Uganda DLT. The first author and four of the co-authors worked at, or closely, with the Uganda MoH over the last two decades, which facilitated the identification of Key Informants and the location of relevant documents, especially those not in the public domain.

The interview guide sought perspectives of respondents regarding their experiences with the DLT development and implementation, assessing the DLT along the attributes of a model HSPA framework, and whether Key Informants considered the DLT successful in achieving intended objectives. All the interviews were carried out by the first author in English, between June and August 2012. At the point of 30 interviews spread over the key constituencies, descriptive saturation was achieved (see Table 2). The interviews were audio recorded, transcribed, coded, and analyzed by the first author. The outputs were reviewed by two other members of the research team. Key Informant responses were analyzed together with information from grey and published literature to inform the critique of the DLT from multiple perspectives. In one approach, inductive analysis was used, and the findings were utilized to relate the story of the development and implementation of the Uganda DLT [17]. In the study reported on here, deductive analysis, using the attributes of the normative HSPA framework, was applied to primary KIIs’ data and grey and published literature to provide another perspective to the critique of the DLT. In addition, a quantitative aspect of the critique was carried out, whereby quantitative data from the DLT database was analyzed using hierarchical cluster analysis [18].

**Table 2:** Key Informants Affiliation and Responsibility

Institution		Code
National Level	Ministry of Health	MOH 1
		MoH 2
		MoH 3
		MoH 4
		MoH 5
		MoH 6
		MoH 7
	International Agency	IA1
		IA2
		IA3
	Academia	ACAD1
		ACAD2
Local Governments	Political Leaders	DPOL1
		DPOL2
	Administrative Managers	DADM1
	Technical Managers	DTECH1
		DTECH2
		DTECH3
		DTECH4
		DTECH5
		DTECH6
		DTECH7
		DTECH8
		DTECH9
		DTECH10
		DTECH11
		DTECH12
Civil Society Organisation		CSO 1
		CSO 2
		CSO 3

*MOH* Ministry of Health, *IA* International Agency, *ACAD* Academia, *DPOL* District Politician, *DADM* District Administrator, *DTECH* District Technical Officer, *CSO* Civil Society Organisation;

## Results

The findings of this study are presented here in three sub-sections: (1) highlights of the Uganda health system context over the last two decades; (2) the introduction and implementation of the DLT; and (3) a review of the DLT along the seven attributes of the normative HSPA framework.

### The Uganda health system context, the mid- 90s to date

Since the mid-90s, Uganda has implemented a number of generic and health system reforms. This was in the context of recovering from several years of political and armed conflict. At the generic level a number of governance reforms have been implemented including decentralization, and a return to multiparty democracy [19]. Uganda’s decentralization reform has been cited as one of the most radical devolution programs in LMICs [20]. The 1995 Constitution and the Local Government Act 1997 form the basis for decentralization [11, 12]. Uganda’s Constitution states *‘the state shall be guided by the principle of decentralization and devolution of government powers to the people at appropriate levels where they can best manage and direct their own affairs’* [11].

**Fig. 1:**
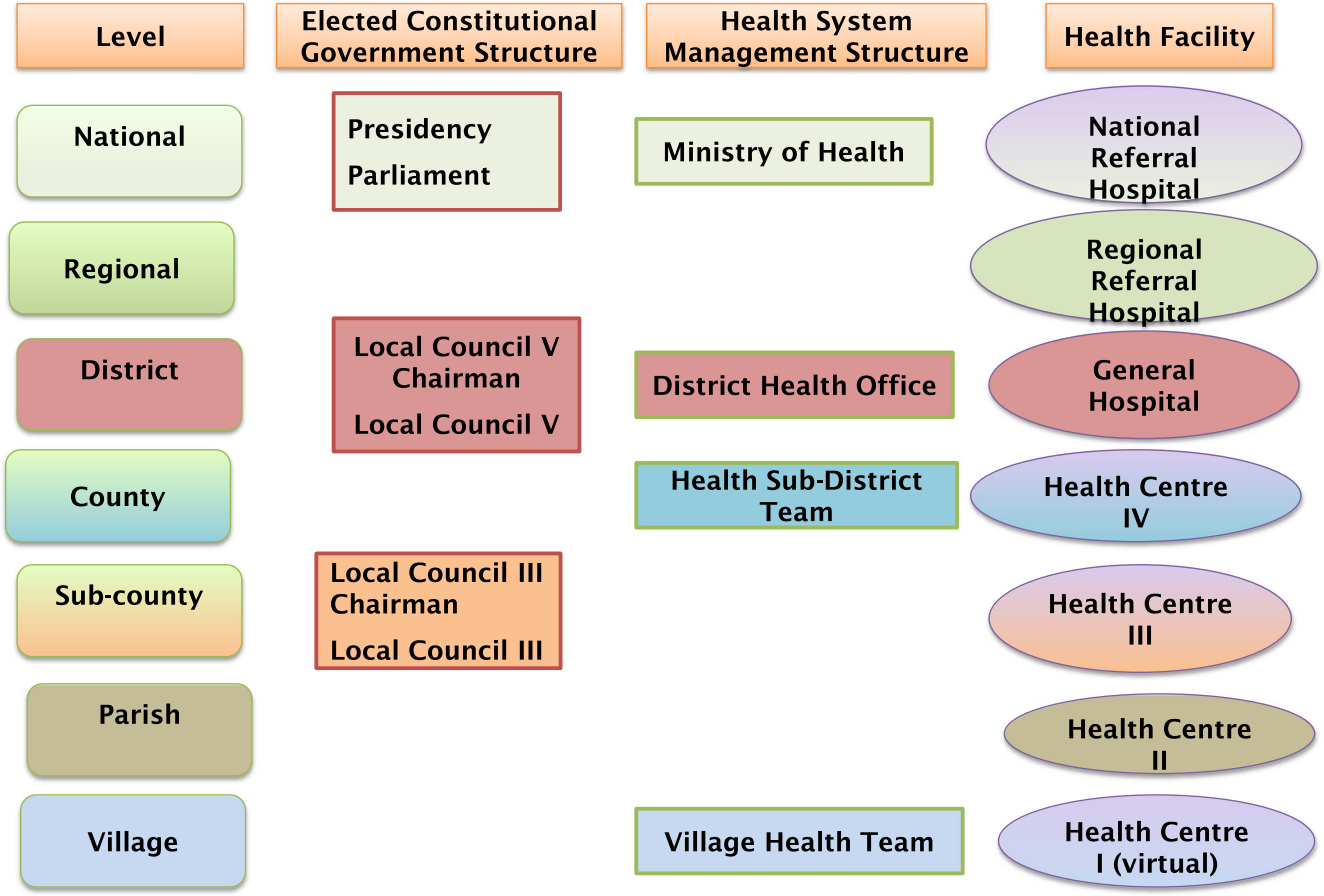
The relationship between political, health system management and health care delivery system structures

In the health sector implemented reforms include sector wide approach to health development (SWAp) and Public Private Partnership for Health (PPPH); and financing reforms including introduction and subsequently abolition of user fees, and the use of the government budget as the main channel for providing public and donor resources for the health sector [17]. SWAp was associated with joint planning among major health system stakeholders, channeling of the bulk of development partner funds though the national budget (budget support), and joint monitoring of sector performance [20–22]. User fees in public facilities were introduced across the country beginning from the late 80s, in a context of very low funding for the health sector and with the encouragement of some of the international agencies. The User fees were collected and retained at the health facility. However, user fees were abolished by the country’s leadership in 2001 [23]. PPPH policy documents were drafted, representatives of the private sector participated in health system planning and coordination structures and Private not for Profit (PNFP) facilities benefitted substantially from the public health sector funding [24]. Uganda health system stakeholders sought to adapt the generic decentralization reform to the health sector. This involved the elaboration of sector structures at the sub-national levels, and the elaboration of the package of services to be delivered at the different levels. The structures of government (political), health system management and health care delivery in Uganda are closely related as shown in Fig. 1 [17, 25].

Since the mid-2000s another set of changes have taken place. Global Health Initiatives (GHIs) were introduced in the mid-2000s to support attainment of Millennium Development Goals (MDGs). GHIs including the Vaccine Alliance (GAVI), the Global Fund to fight AIDS, Tuberculosis and Malaria (the Global Fund), and the President’s (United States of America) Emergency Plan for AIDS Relief (PEPFAR), have supported the country significantly, providing the bulk of development partner funding to the Uganda health system over the last decade. In contrast to the financing arrangements under SWAp, the GHIs do not provide ‘budget support’, but rather provide funds and inputs for health services of interest, directly to programmes and implementers at the national, district and health facility levels [26]. Since the mid-2000s, public funding to the health sector stagnated especially for decentralized health services affecting both public and PNFP health services providers. The resulting health system financing landscape is characterized by wide variations in funding for districts, coupled with limited information on development partner funding to the individual districts [17].

Additionally there have been a number of changes in regard to how decentralization is implemented in Uganda in the last decade. There has been a marked increase in the number of districts, from 39 in 1993, to 56 in 2003, and 112 in 2010. An additional 23 districts were approved by Parliament and are to be operationalized between 2016 and 2020. A mix of, recentralization of some of the functions previously carried out by district managers, and retention of mandates expected to be devolved to the local governments by the national level, has been noted over the last decade. A mixed approaches model for the purchase and distribution of medicines was practiced in the early 2000s, the push-pull medicines reform, whereby districts played a key purchasing role. This approach however was disbanded in 2009, and the responsibility for medicines purchasing and distribution was returned to the National Medical Stores [27, 28]. In the mid-2000s the Ministry of Local Government introduced the Fiscal Decentralisation Strategy, whereby the districts were allowed to reallocate sector conditional funds to local priorities. This was in the context of more than 80% of funds received by the districts from the national budget being in the form of sector conditional grants, which they had to spend against specific guidelines. Local revenue over which the district managers had leverage on average contributed less than 10% of district health system budgets. However, the Fiscal Decentralisation Strategy was opposed by priority sectors that controlled the conditional grants, including the MoH [29]. In regard to human resources management, staff norms, budgets for recruitment of new staff, magnitude of staff salaries, and even which cadres to recruit, are determined at the national level by the Ministries of Health, Finance and Public Service [17].

### The Uganda District league table

The Uganda Annual Health Sector Performance Report (AHSPR) was introduced to provide a comprehensive report on sector performance for all the health system stakeholders in line with joint monitoring espoused by the SWAp. The first AHSPR was produced in 2001. Given decentralization, need was identified to assess the performance of individual districts, and the DLT was introduced in 2003. The objectives of the DLT were: comparing performance of districts to determine good and poor performers; providing information to facilitate understanding of good and poor performance thus enabling application of corrective measures; increasing local government ownership of achievements; and encouraging good practices. The DLT was composed of a number of input, process and output indicators as shown in Table 3. The DLT was based largely on the Health Management Information System (HMIS) and included data on public and PNFP health facilities across the country. Household latrine coverage (a proxy for sanitation) was compiled from community surveys, and input and management indicators were distilled from administrative records.

**Table 3:** Uganda District League Table Indicators and Weighting Factors

Indicator	Year	Weight factor	Year	Weight factor
*Introduced in 200*3				
Population				
No. of health sub-districts				
No. of hospitals				
No. of health facilities				
Public health funding per capita				
Approved post filled by qualified health workers	*200*3*–05*	*5*	*2011-*	*10*
HMIS outpatient returns submitted timely	*200*3–10	*5*		
HMIS outpatient returns submitted complete	*200*3–05	*5*		
PHC funds spent on medicines and supplies at NMS & JMS	*200*3–10	*10*		
Quarterly funding requests submitted timely	2003–05	*5*		
Children < 1 received DPT third dose as per schedule (DPT3)	*200*3*–10*	*12.5*	*2011-*	*15*
Government and PNFP OPD utilization per capita	*200*3*–10*	*12.5*	*2011-*	*10*
Household pit latrine coverage	*200*3*–10*	*7.5*	*2011-*	*10*
Deliveries in government and PNFP health facilities	*200*3*–10*	*12.5*	*2011-*	*15*
Proportion of TB cases notified compared to expected	*200*3*–10*	*10*		
Pregnant women receiving second dose of Fansidar for IPT (IPT2)	*200*3*–10*	*10*	*2011-*	*5*
*Introduced in 2006*				
PHC funds disbursed that are expended	*2006–10*	*5*		
Fiscal Decentralisation Strategy (FDS) flexibility gain	*2006–10*	*5*		
HIV/AIDS services availability composite (ART, PMTCT, HCT)	*2006–10*	*10*		
*Introduced in 2011*				
HIV testing of children born to HIV positive women	*2011-*	*10*		
Antenatal care 4^th^visit	*2011-*	*5*		
TB treatment successrate	*2011-*	*5*		
HMIS reporting composite (completeness & timeliness)	*2011-*	*10*		
Medicines orders submittedtimely	*2011-*	*5*		

Source: MoH 2003; 2006; 2011; Tashobya et al. 2015;

*HMIS* Health Management Information System, *PHC* Primary Health Care, *NMS* National Médical Stores, *JMS* Joint Medical Stores, *DPT* Diptheria Pertussis Tetanus, *PNFP* Private not For Profit, *OPD* Out patient Department, *IPY* Intermittent Presumptive Treatment, *ART* Anti-retroviral therapy, *PMTCT* Prevention of Mother to Child Transmission, *HCT* HIV Counselling and Testing, *TB* Tuberculosis, *HIV* Human Immune-deficiency Virus, *AIDS* Acquired Immune Deficiency Syndrome;

The process of producing the DLT was initially led by the Health Planning Department in collaboration with the Resource Centre, and other technical programmes of the MoH. District data was compiled, and analyzed by weighting some of the indicators and ranking the districts from the ‘best’ to the ‘worst’ performer using the resulting index. Categorization was done, with the designation of the ‘top 10’, ‘middle performers’, and bottom 10 districts. The ‘top performers’ were recognized in public fora and the ‘bottom performers’ advised to improve [18]. The AHSPR including the DLT were some of the key documents presented at the Joint Review Mission and the National Health Assembly, once a year and once every 2 years respectively, the key fora for sector consultations in the framework of SWAp. The Joint Review Mission includes representatives of the MoH, other relevant ministries, representative of development partner agencies, the private sector and selected district political, administrative and technical managers. The National Health Assembly is a bigger forum including all those attending the Joint Review Mission plus political, administrative and technical managers from all the districts of the country.

Two main adjustments have been made to the DLT, coinciding with the development of new sector strategic plans in 2006 and in 2011, (Table 3). A number of indicators were dropped and some new ones introduced, and some changes were introduced in the weighting factor of some of the indicators. In 2011 sub-groups of districts were explicitly introduced into the analysis, and the Kampala City Council Authority was treated in a special way due to the recognition of its peculiar status (urban character, many referral health facilities). The number of districts singled out for particular mention at either end of the performance spectrum increased from 10 to 15 given the increase in the number of districts [18].

### Review of the DLT using a normative HSPA framework

Below are the findings of a critique of the DLT along the seven attributes of a normative HSPA framework**.**

#### Process of development of the HSPA framework

The study noted that a range of stakeholders were involved in the development of the DLT: technical officials from the national level including officials from various departments of the Ministry of Health (MoH), and representatives of development partners, the private sector and civil society. A few district technical officers were involved, but not the political or administrative managers.

“*Reflecting back on how the DLT started – it was technical people at the MoH with the participation of a few districts*” Academia (ACAD) 1

“*The DLT was championed by the MoH and led by the Health Planning Department. I am not sure about the involvement of local governments in the development of the DLT. I think the role of the local governments was really limited. I think it was not inclusive especially of the people to be assessed*” District Technical Officer (DTECH) 4

Other groups of health system stakeholders that were noted not to have been involved are researchers and those responsible for generic data collection and performance assessment. The Uganda Bureau of Statistics carries out censuses, demographic health surveys and panel surveys on key health system issues. The Ministry of Local Government is responsible for generic local government performance assessment, whereas the Office of the Prime Minister is responsible for overall national performance assessment. The individuals who participated in the development of the DLT were mostly biomedical and public health/statistics professionals, with hardly any social science/organizational management professionals. There was no evidence of utilizing data/evidence for pointing out causal links between different variables of the DLT and justifying the league table approach as the model of performance assessment to be used [18].

#### Relationship with the health system framework

The second attribute refers to the need for the HSPA model to be *embedded in an explicit health system framework*. The analysis of interviews and relevant documents noted that over the previous two decades the National Health Policies (NHPs) and Health Sector Strategic Plans (HSSPs) have articulated a distinct conceptual framework of the Ugandan health system [25, 30–34]. The more recent strategic plans are supported by a Monitoring and Evaluation Framework, which details how HSPA should be approached at the different levels of the health system [35]. The DLT reflected different components of the NHP and HSSPs with focus on key sector priorities.

“*The DLT was embedded in an explicit health system framework as presented in the NHP and HSSP”* Ministry of Health Official (MoH) 2

However, ambiguity was noted in the relationship between the HSPA framework and the conceptualization of the Uganda health system. The NHPs and HSSPs portray the health system in its broad sense, as reflected in the World Health Organization (WHO) definition of a health system as ‘*the sum total of all organizations, institutions and resources whose primary purpose is to improve health’* [1, 34, 35]. The more recent sector strategic plans highlight the major contribution made by social determinants of health (SDH) and recognize various institutions and organizations as key stakeholders in the health system. These include entities like the ministries responsible for Finance, Agriculture, Public Service, Education, and private health services providers [34]. However, the contribution of such entities is largely not reflected in the structures and frameworks for HSPA. The DLT, with the exception of the indicator on household latrine coverage, didn’t cover aspects of the broader health system that affect population health. The respondents in the study noted that the DLT was limited to health care outputs, and did not extend to health outcomes.

“*The multi-sectoral nature of health though does not come out clearly. … the wider variables – education, roads, but these are necessary for analysis at local government”* DTECH 4

*“The LT’s very design is restricted to what the MoH and the DHO* (District Health Office) *is doing and even then it is restricted to what is measurable, through a tool that is available, that is the HMIS. Therefore anything that is not amenable or cannot be measured is not included” DTECH* 1

In addition to improvements in people’s health, the Uganda health sector documents have indicated other health system goals, specifically fair financing for health [33]. The DLT however did not provide for the tracking of such goals.

“*Our system goals are towards better health, financial risk protection, social justice and equity. The DLT is an intermediate step. I do not think we took it a step further. May be there was a gap. There should have been a second step*” International Agency (IA) 1

#### Relationship with the policy, organizational and social context

The third attribute indicates that the HSPA framework should *relate to the policy, organizational and societal context in which it is established*. The views of the respondents of the study, and the various documents that were consulted, indicated that the DLT was seen as relevant to the Ugandan health system context, especially at the time of its introduction. The initial implementation of decentralization provided the policy and institutional framework for district health system functionality; whereas SWAp and PPPH supported functionality of a coherent health sector. The multiple reforms worked synergistically to support integrated health services delivery within the district, and provided a conducive environment for system-wide performance assessment at national and sub national levels [17, 36].

“*The DLT is appropriate, answering to the context of decentralization*” MoH 4

“*There is power and decision-making at the district – so this makes the DLT appropriate”* DTECH 24

Over the last 14 years since the introduction of the DLT however, a number of changes have taken place in the Ugandan health system context with implications for decentralized and integrated health service delivery and HSPA. The joint planning and common funding arrangements previously practiced under SWAP no longer applies. The current scenario of limited public funds, with significant funding from GHIs on which there is limited information and poor predictability of disbursements to individual districts and implementing entities, presents challenges for effective decentralized health services delivery and HSPA. The indicator on the DLT on magnitude of funding refers to public funding only.

*“With the Global Health Initiatives it becomes rather complicated. HIV/AIDS service delivery for example, may not be a district thing – a high proportion of HIV/AIDS funding comes from donors. And there are some variations; for example, there was thinking that West Nile (region) had low levels of HIV and did not require support” DTECH2*

*“There is fragmented funding for the district. With minimal public funding and mainly partners who fund districts directly. It is very difficult to get information about this funding” MoH2*

The marked proliferation of districts has led to smaller districts in terms of surface area and population, and stretched the health system management capacity. At the same time there has been high turnover of health system managers, with many of the experienced ones seeking employment amongst the GHI supported agencies. Many of the health system managers in the new districts had limited prior management experience [17].

*“There have been many changes in the context; there are many new districts, the capacity of district managers is questionable, and resources are spread too thin”IA3*

The failure to shift more responsibilities to the district level in line with the mandate provided by decentralization, and in some cases recentralization of some functions as has been noted, has created challenges to district health systems management and HSPA. In practice, district health system managers do not have room to make major decisions on health services delivery. The indicators that were intended to assess management processes have changed frequently, largely reflecting the changes in context. Examples of this include the indicators relating to the Fiscal Decentralisation Strategy and the proportion of the PHC budget used to purchase medicine at the NMS and Joint Medical Stores (JMS).

The governance reforms of decentralization and multiparty democracy were intended to ensure participation of the community in decision making at the different levels and support accountability in regard to provision of social services to the population. Political, administrative and health sector specific structures have been put in place to support these processes. However despite the existence of these structures, there has been limited involvement of members of the community in HSPA [17]. The DLT is discussed at national level; it is expected that the DLT and other outputs of the HMIS are discussed at the district level among the political, administrative and technical managers. However the practice varies markedly across the country.

*“I do not get the sense that people go back and ask, ‘why was I in this position’”* District Political leader *(DPOL) 2*

The private sector in Uganda provides a substantial proportion of health services, and manages a significant portion of the expenditure on health, especially resources from the households and development partners [10]. The services delivered by the facility-based PNFP are captured in the DLT. However most of the services provided by other private health services providers including non-facility based PNFP providers are not captured. The funds managed by the private sector are not captured in the DLT.

### Elaboration of the HSPA framework

The fourth attribute states that the HSPA framework *should be well developed with a conceptual model, clear purpose, dimensions, sub dimensions and indicators.* The objectives of the DLT were clearly articulated at the time of its initial development and have been maintained since. The objectives, are a combination of aspects of accountability of the districts to the national level (comparison between districts, determining poor and good performers); and support for decision making at the national and local government levels (understanding factors behind observed performance, encouraging local government ownership and learning from good practices) [13, 18]. The DLT is composed of indicators reflecting system-wide and programme specific performance, covering inputs, processes, and outputs (Table 3). The DLT however, was neither based on an explicit conceptual model, nor did it have designated dimensions relating the indicators to one another. The league table provides a comparison across the districts assessing the performance of each district on the basis of individual indicators, and a composite index computed by weighting the indicators. With the exception of the objectives and indicators of the DLT which were documented in the various AHSPRs, there was minimal documentation of the DLT.

Respondents in this study were of the opinion that the initial choice and range of indicators were reasonable. These indicators were derived from sector strategic plans and the choice of those to include in the DLT was influenced by the availability of data.

“*I think we covered the health system building blocks and the priorities within the sector along the lines of the MDGs- child health, HIV/AIDS, malaria, TB*” IA 2

Over time health system stakeholders have raised concerns that the DLT input and process indicators were not adequate to facilitate analysis of the factors underlying observed performance at district level [18, 37]. In view of this, and given the changes in context, a number of process indicators were dropped and new ones introduced in 2006 and 2011 (see Table 3). However a number of the respondents were of the opinion that this aspect of the DLT could be improved on.

*“In reviews of sector performance, management has been noted as having a major role and was lacking on the DLT. The management indicators are challenging for example supervision which is important is difficult to measure or monitor*” MoH 4

“*There are two categories of indicators: those to do with the coverage and quality of care making up 75% of the league table score; and those on management contributing 25%. We can blame everything else but if the leadership and management are poor, these things will not happen. I think at some point we need to say that if the issue is management why don’t we give it a bigger score and then we asses that*” DTECH 12

District health system managers have raised concerns that their leverage on some of the indicators that were used to assess their performance on the DLT, and which were included in computing the ranking index, was limited. Such indicators include household latrine coverage and the proportion of approved posts filled by qualified staff [38].

*“Even as a district manager it is true you can have an influence on it (*human resources establishment*) but sometimes you may not. For example there is now a ban on recruitment*” DTECH1

DLT indicators that were deemed to score poorly against technical criteria for quality of performance indicators were replaced in 2006 and 2011. There were no indicators for non-communicable diseases, as these were not recognized amongst sector priorities by then [30, 31]. The DLT was composed of quantitative indicators; it did not provide for collection of qualitative data. This was considered a major omission by some of the respondents.

*“As the DLT on its own is mostly based on statistical data focusing on coverage and outcome indicators …we found that information was not enough to facilitate detailed analysis.”*MoH4

#### Institutional set-up for performance assessment

The fifth attribute requires that the framework should be supported by an appropriate *institutional set-up for performance assessment*. This attribute covers policy and institutional provisions for HSPA, data availability and quality, and existence of champions and networks that bring together HSPA stakeholders. Responsibility for HSPA, and specifically for the DLT, is shared between MoH, districts, heath sub-districts and health facilities. HSPA is a shared responsibility at the MoH, between the Resource Centre, the Quality Assurance and Health Planning Departments. However, there is lack of clarity on who holds the responsibility for data analysis, packaging and presentation in support for evidence-based decision making. A restructuring exercise of the MoH carried out in 2009 introduced a Monitoring and Evaluation Division within the Quality Assurance Department which was supposed to be responsible for these functions; however it has never been functionally constituted [39]. DLT computation and publication have oscillated between the Resource Centre, and the Health Planning and Quality Assurance Departments, depending on the managers’ and individual officers’ interest and capacity for HSPA. Some of the technical programmes, like the Expanded Programme for Immunization, and the AIDS, malaria and tuberculosis control programmes, have with the support of development partners developed parallel systems of reporting including comparing performance across districts. Some of the respondents though were of the opinion that some aspects of HSPA should not be housed at the MoH, given its many other responsibilities.

*“This (*compilation of the DLT*) should not be within the work of the MoH – there are too many other things. There is need to create specific institutional capacity for health system performance assessment”* ACAD 1

The bulk of the information used for HSPA in the sector, including for the DLT, is generated from the HMIS. The HMIS was introduced in Uganda in the mid-90s as a paper-based system which has benefited from modest, piecemeal investments in human, financial and technological resources over the years. In 2012 the HMIS was converted to an e-HMIS [40]. Reviews of the HMIS are carried out every 5 years; data validation is carried out on ad-hoc basis [39]. There are challenges in the quality of data for HSPA, including for district HSPA. HMIS data validation exercises have highlighted substantial differences for some districts between data at health facilities, district databases and data submitted to the MoH Resource Centre. There are gaps in the timeliness and completeness of reporting by the health facilities [41]. Data on a number of indicators, especially the ones pertaining to district health resources, has not been routinely available in the DLT over the years. Where such data is available, it is usually relating to the public resources availed through the government budgeting and planning system, but does not include resources from development partners and from the private sector including the PNFP sub sector and the direct contribution of households [18].

“*Which establishment (*for human resources*) are we looking at? Is it one of the government or government plus PNFP? Because the performance reported (in the DLT) covers government plus PNFP and we are looking at this indicator because it influences performance” DTECH1*

Another challenge is the lack of regular and reliable district health outcome data. This includes data on health outcomes like infant, child, maternal and adult mortality; contraceptive prevalence; fertility rates; nutritional status; and HIV prevalence. Data on these variables is only available from the demographic health surveys and other population surveys, which take place once every 3 to 5 years, and for which data is aggregated at regional level. There is no government at the regional level. Uganda lacks a functional vital registration system; even health facility deaths and births are not linked to districts and sub counties of origin [39].

Limited use of HSPA data, including the DLT data, for decision-making was noted. This was largely attributed to capacity gaps at the different levels of the health system, and minimal interest. Sub national units including districts and health facilities operate largely as data sources and conduits and less as users of data for decision making. There are marked gaps in human, financial and technological resources for HSPA at all levels of the health system including the national, district and health facilities [39]. Interest in HSPA at district level tends to be patchy.

“*My main training was in health management so I am a little bit different from the other district health managers. If the district did well in some circumstances but did not do well in others, we could look for reasons why. ..Whom are we having in leadership?”* DTECH 12

The study noted limited use of the available HSPA networks at district, sectoral and multi-sectoral level. For example, the Supervision, Monitoring and Evaluation and Research Working Group that was put in place as a sector forum for HSPA at the national level does not seem to have had a significant impact on Uganda HSPA [39]. There have hardly been any efforts to link district system wide performance assessment with programme performance assessment initiatives. The quarterly requirement for sectoral reports by the Office of the Prime Minister is no more than a compilation of data on a number of sectoral indicators. The lack of champions for HSPA has been indicated as a challenge in the Ugandan health system [17].

#### Mechanisms for eliciting change in the health system

The sixth attribute of a HSPA framework is that it should *explicitly indicate mechanisms for eliciting change in the health system*. Aspects of this attribute relate to the compilation, analysis, and presentation of HSPA findings; the existence of fora for discussion of such findings; and the actual mechanisms through which the information provided is expected to lead to changes in the health system (theory of change). Compilation and analysis of the DLT is carried out by technical officials of the MoH. The league table is published on annual basis, with data on a number of indicators (Table 3), for each of the districts, and a composite index, to rank the districts from the ‘best’ to the ‘worst’ performer. Rationale for the application of different weights to the indicators in the computation of the DLT rank is not explicitly documented and can only be assumed from statements in some MoH documents [18].

The analysis as provided by the DLT has been criticized as inadequate and inappropriate, especially by district health system managers. They argue that districts face contextual and structural differences, and should not be compared across the board as is done by the DLT, without taking into consideration the differences [38]. The marked increase in the number of districts over the last decade has made the league table unwieldy, and as a result many stakeholders tend to only focus on the district DLT rank. The highlighting of the top and bottom 15 districts leaves 81 districts as middle performers, without clear recommendations [18]. Since 2011, the districts are categorized into smaller groups according to: the date of creation, the size of population, and the perceived extent of disadvantage [35]. However the analysis related to this categorization was deemed as inadequate by some of the respondents.

“*The DLT was intended for the review of district performance, sharing of experiences, both good and bad, and as a peer review mechanism. This however requires comparison of like and like, consideration of absolute versus incremental performance and improvements, and taking into consideration the multi-dimensional perspectives of health*” DTECH 4

The DLT is presented as part of the AHSPR at the Joint Review Meeting and the National Health Assembly. These meetings are usually 2 to 3 days of intense activity. The time allocated to HSPA, including the DLT, and the depth of discussion on it, leave a lot to be desired [42]. Other dissemination modalities of the DLT are limited.

“*The fora for discussing the DLT findings are appropriate but not adequate. The Joint Review Mission and National Health Assembly – the wide stakeholder representation is good. However time is not adequate for meaningful discussions because the agenda is broad. It has been proposed that these discussions should go to the regional leve*l” MoH 4

The study did not come across any documentation of the envisaged mechanism(s) on how the DLT was expected to effect change or influence decision-making in the Uganda health system. The study noted that some decisions have taken place in the Uganda health system as a result of DLT data: MoH supervision teams have used DLT information for the purpose of support supervision; MoH and some of the districts have used the information for improving planning and management practices; development partner organizations have used the DLT information in determining districts to provide support to [37, 43, 44]. The mechanisms for change that are noted to have been at work (implicit) are benchmarking and utilization of quality improvement initiatives. Conversely, there were unintended effects of the DLT. Although it was indicated that the DLT was not intended to “name and shame”, it has been reported to have caused embarrassment and resentment among managers of districts portrayed by the DLT as performing poorly [17, 38]. The limited use of DLT information for decision-making has contributed to decreased interest in the DLT at all levels, and especially at the district level. In more recent years the DLT has been seen more as a ritual than an aid to decision-making [38].

“*There is no explicit decision or policy that has come out of the DLT in the last 10 years. There has not been much incentive – it is just being in the top 15. It should be more than this. There is no attempt to link the different indices within the DLT. Why are we doing poorly on a certain indicator and well on another? The way it is, the good practices do not come out clearly. The DLT is very much examiner/examinee interface. There is a lot of listening to be done by the local governments and limited discussion*” DTECH 9

“*Depending on what is underlying poor performance it may be difficult to address even by the MoH. Where for example there are poor management practices, leadership that is not encouraging teamwork or delegation, those can be emphasized through supportive supervision by the center, through the Area Teams. Resources are a little difficult e.g. if a new district is provided with a vehicle they are able to reach facilities for support supervision. If that is not done the district will remain lagging behind. You find that in some of the districts that do not come out of the bottom five*”DTECH1

#### Adapting to change in context and time

The seventh and final attribute refers to the *adaptability of the framework to different contexts and over time*. The Uganda health system context shows variation across the districts, more so now with the increased number of districts, and over the last 14 years the DLT has been in use. This study noted that some efforts were made to adjust the DLT taking into consideration some of the changes in context and to bring on board new thinking on HSPA. The changes that were made were in regard to dropping existing and introducing new indicators, at the process/management and output levels.

*“Most of the (management) indicators were obsolete; we were trying to look for new ones”* MoH6

The DLT objectives and the main approach to performance assessment i.e. the league table ranking were not changed. There was no provision of different application of the model across the country.

## Discussion

In this section we consider the findings from the review of the Uganda DLT using a normative HSPA framework in light of available literature and experiences, for the purpose of supporting the development of recommendations for updating the Uganda district HSPA framework, and to tease out lessons for LMIC and global researchers and policy-makers.

The study noted that during the *processes of development and adjustment of the DLT,* the stakeholders that were involved were mostly from the national level especially the MoH and development partner representatives most of whom were biomedical or public health/epidemiological professionals. Experiences from other countries have shown that bringing on board a wide range of stakeholders including a mix of policy makers, data collectors and data users; a range of professions and sectors, contributes to the appreciation of the multi-sectoral and multi-faceted nature of HSPA and improves the likelihood of using resulting data for decision-making [7, 45]. The restricted involvement of stakeholders in the development of the DLT is likely to have contributed to the limited understanding and ownership of the DLT. It is the recommendation of this study that in future processes of development and/or adjustment of the Uganda district HSPA framework should involve a wider group of stakeholders with particular emphasis on district technical, political and administrative managers; researchers; representatives of various entities that collect and use data; and include individuals with different professional backgrounds including the biomedical, public health/statistics, and social science/organizational management.

The use of evidence, and explicit models indicating causal links at the time of development of the framework, it has been shown, improves stakeholder confidence and buy-in [4, 5]. In the case of the Uganda DLT there was no evidence of the use of data and/or modeling during the development and adjustments, which omission may have contributed to the criticism of the model by some of the stakeholders. It is recommended that in future efforts to develop/adjust the Uganda district HSPA framework data is used to justify models that are being used.

A HSPA framework should reflect the understanding that health system managers are directly responsible for the management of the health care system, and indirectly for other aspects of the health system as health system stewards, and thus facilitate performance assessment across the health system [1]. This study has shown that there is a gap in sub-national performance assessment in Uganda as embodied in the DLT, between the *health* system and the *health care* system. This is not an isolated finding; the lack of clarity on how the health care system relates to the other aspects of the health systems has been noted globally, and especially in regard to health system performance assessment [3, 14]. However, lessons can be learnt from how some HSPA frameworks have been structured to address some of these challenges. The Canadian Health Indicator Framework for example was based on the Lalonde model of the health system which highlights the non-healthcare determinants of health. The Dutch HSPA framework builds on both the Lalonde model and the Balanced Score Card to support the explicit indication of how the healthcare system relates to the broader health system [3]. It is the recommendation of this study that the Uganda district HSPA framework should be clearly situated in the wider national health system and HSPA framework that recognizes SDH. The HSPA framework should explicitly lay out the expectations from the health care system and other sectors, and thus facilitate the MoH and DHO as they seek to hold the different sectors accountable for actions in their domains. The framework should highlight the aspects of the HSPA framework where the MoH and the DHO are directly responsible and the aspects where they track progress as stewards.

A HSPA framework should be responsive to the context in which it is situated [3, 7]. The Uganda policy, organizational and social context is very complex, and dynamic. The DLT assumes a coherent organization in terms of priority setting, management of resources and performance assessment. To a large extent this was the prevailing situation in Uganda in the early 2000s, with the early implementation of decentralization and SWAp. However over the last decade there have been a number of changes in sector coordination and funding that negate this scenario. Additionally, the proliferation of districts, stretched health system management capacity and retention and/or recentralization of some of the functions that should have been at the sub-national levels have contributed in practice to limited decision-space at the district level.

With the DLT, data on district performance is submitted to the national level, whereby it is analysed and presented, with the purpose of supporting decision making at the national, district and lower levels. Particular emphasis is put on comparison of performance between districts across the country. The DLT takes an (upward) accountability approach, which assumes good information on resources available to the districts at the national level and much more leverage than is the case today. It is the recommendation of this study that a different approach to district HSPA should be taken. This new approach should emphasize the collection, analysis and use of data for decision-making at the district level. This is in recognition that the district level has better access to information on resources available for service delivery including human and financial resources, and the detail of operational information. Such an approach we argue will provide for a more conducive environment for inter-sectoral collaboration at the district level and ownership of health system performance by the political, administrative and the technical managers – beyond the DHO. Quantitative and qualitative indicators should be built into such a tool. Such a tool would facilitate learning at the district level. Despite the observation that decision-space at the district level in Uganda is limited, opportunities do arise which empowered managers can take advantage of to improve district health system performance. Appropriate district HSPA tools can facilitate such managers. The role of the national level would be to develop appropriate models for district HSPA, to support districts in applying these models and to build capacity for HSPA.

Uganda has put in place the legal and institutional framework for decentralization and multiparty democracy. However the promises of these reforms in regard to participation of the community in decision-making and enhancing accountability are yet to be achieved. The low capacity of the population to demand for accountability has been related to the poor levels of socioeconomic development and to the history of conflict [46, 47]. The DLT did not facilitate the link between the health system and the community it serves for purposes of HSPA. There are some promising experiences of civil society organizations working with communities to improve their capacity to engage with the government in regard to demanding for accountability and pushing for improvements in service delivery [48, 49]. It is the recommendation of this study that efforts are taken to learn from such examples and to develop mechanisms for providing accountability to the communities that are served (downward accountability). Civil Society Organizations have been noted to be better at such innovations and can utilize both formal and informal structures for the purpose. The new/adjusted model of Uganda district HSPA should be set up to link with such innovations.

Theoretical and empirical studies have highlighted the importance of having a well-documented HSPA framework, with a conceptual reference model, clear purpose, dimensions and sub-dimensions and performance indicators [3, 50]. The lack of a conceptual model and designation of dimensions and sub dimensions made it difficult to appreciate how the DLT interfaced with the wider health system, and how the different aspects of the DLT interfaced with one another. Over the last 14 years attempts were made to improve the quality of the DLT indicators in line with what various scholars have indicated as desirable characteristics for performance indicators [7, 51]. However, gaps and challenges still exist, particularly concerning the lack and/or inadequacy of indicators related to non-health care determinants of health, input and process indicators, and indicators pertaining to the management of non-communicable diseases. Lessons can be learnt from a number of countries that have implemented HSPA frameworks over time, whereby indicator lists evolve depending on the health system information requirements and the capacity for data management [16]. This study recommends that a conceptual model should be elaborated for Uganda district HSPA clearly linking it with the broader health system, and highlighting dimensions and sub-dimensions. The objectives of the district HSPA should be reviewed with the view to make sure this relates appropriately with the current context and the data that is being collected. A strategic approach should be taken towards the evolution of the performance indicator list, starting with those that are most needed to support decision making at the district level. Our recommendation is that emphasis in the short term should be on developing/adapting good indicators for inputs, processes and for non-communicable diseases.

A number of researchers highlight the importance of the institutional set-up for the implementation of any HSPA framework [3, 52]. Uganda has made headway in developing an institutional set-up for HSPA including articulating a Monitoring and Evaluation Framework and steady progress towards a functional HMIS. However major challenges exist in regard to the institutional set-up for HSPA. The absence of an explicit unit for data analysis and packaging at the MoH is likely to have had an influence on the evolution and poor ownership of HSPA and the DLT. Lessons can be learnt from the development and implementation of national HSPA frameworks across the world. In Canada and Netherlands the explicit investment in networks for HSPA led to learning among the stakeholders and highlighted the comparative advantage of the different entities. In Australia senior political and generic administrative managers were utilized as champions for HSPA which helped to emphasize the multi-sectoral approach [3]. In South Africa a private company the Health Systems Trust has for several years been responsible for the analysis and presentation of district HSPA in the form of the District Health Barometer [16]. This study recommends that an explicit unit should be indicated in the Ugandan health system and appropriately facilitated, to support HSPA across the country. Such a unit would focus on carrying out data analysis and presentation; providing models for data analysis and presentation at sub-national levels including the district level; and facilitating the development and functionality of champions and networks for HSPA.

Questions have been raised about the validity of the conclusions of the DLT given the quality of data [17, 41]. The strategic approach to improvements in district HSPA earlier highlighted should be extended to improvements in the quality and range of data. It is the recommendation of this study that key stakeholders should agree on data requirements for district HSPA in the short, medium and long term and plans made on how to get the data, taking into consideration available government and development partners technical and financial resources. System-wide data quality assessments should be held regularly – at a minimum in selected districts annually, and across the country every 2 to 3 years.

The analysis of data and the presentation of the information produced in HSPA affects its use for decision-making [7, 50]. The main approach to data analysis and presentation of the Uganda DLT is the league table approach. The capacity of the DLT to present many data points relating to the different districts and indicators, including ranking using a composite index, makes it a convenient tool. However there have been a number of concerns raised with the use of the DLT in Uganda which include: comparing entities which are not comparable; the use of a summary rank that is difficult to interpret; and the difficulty to relate provided information to decision-making. The MoH has acknowledged the concern of comparing districts with marked differences, and since 2011 provides categories within the DLT. However the extent of application of this approach is to list the different districts under these categories with no explicit effort to carry out any further analysis. This study recommends that alternatives/complementary approaches for the analysis and presentation of district HSPA data be sought in addition to the league table. In the quantitative component of the broader research programme this study is situated in, it was demonstrated that hierarchical cluster analysis (HCA) can be used to group districts with similarities, and provide a compromise position between the overly summarized DLT rank and detailed data on all districts and several indicators. HCA also provides an opportunity to look beyond the rank of a district, and to start asking questions about why certain districts’ performance is as observed [18].

The study noted that current dissemination of the DLT in the AHSPR and discussion at the Joint Review Mission and National Health Assembly as appropriate, but inadequate. It is the recommendation of this study that more fora, with emphasis on regional and district level fora, should be sought for discussion of district HSPA findings. Additional opportunities for sharing district HSPA information include quarterly MoH senior management meetings and the various fora at which MoH teams meet with local governments.

Another major gap noted is the absence of an explicit mechanism through which the DLT approach was expected to influence decision-making. Studies in other countries have shown that explicit indication of the mechanisms through which the HSPA framework is expected to cause change is important as it helps to communicate this, manage expectations and evaluate implementation [7]. This study recommends that a theory of change should be explicitly articulated in line with the HSPA framework objectives and other aspects of the framework. Building on previous analyses and recommendations in this paper we propose a district HSPA framework that has as its primary objective the provision of information for decision-making for improvements in health services delivery, and as a secondary objective, comparison of performance across districts. In line with these objectives we would recommend the following theory of change. At the district level the expected mechanism of change should be using district HSPA information for peer learning and implementation of QIIs. At the national level the mechanisms of change should be benchmarking which will inform resource allocation and development of policies and guidelines. At the community level the mechanism of change envisaged is public disclosure of HSPA information. A number of innovative approaches by civil society organizations to support generic and health system performance assessment at the community level have been noted in Uganda [48, 49]. These approaches and opportunities should be further explored and built on.

A HSPA framework should be aligned to its particular context, but at the same time should be adaptable. The DLT lacked dynamism and flexibility across the country. It is recommended that in future development of HSPA provisions should be made to encourage adaptations of the framework across the country, while retaining a core approach that is practiced across the country. Provision should also be made for regular reviews of the framework, say every 5 years, with the view to strategic adjustments as necessary.

### Limitations of the study

Some of the authors were involved in the development and implementation of the DLT. The first author was among the MoH officials that developed the Uganda DLT and were responsible for its early implementation. Four of the co-authors were involved to various extents in the implementation of the DLT. Efforts were made to minimise any bias this may have introduced into the study through a team approach at the various stages of the study including conceptualisation, development of tools, data analysis and report writing. Particular effort was made to involve the researchers that had not been involved in the development and implementation of the DLT at all the stages of the study.

## Conclusion

A review of the Uganda DLT was carried out using a normative HSPA framework. The approach of using a normative model and the Uganda DLT as a case study made it possible draw recommendations for Uganda’s district HSPA and to tease out lessons for global learning. The normative model used in this study can be adapted and used by other researchers and policy makers for the purpose of reviewing HSPA frameworks and/or in the process of developing new frameworks.

The Uganda MoH introduced an innovative district HSPA framework in 2003. A number of achievements and challenges have been accumulated over 14 years of implementation of Uganda’s district HSPA, which provide a base for future developments to build on. The Uganda district HSPA needs to be reviewed by the health system stakeholders and a number of adjustments made. This study recommends that this should be a comprehensive and strategic process, going beyond dropping a few indicators and introducing some new ones as has been done in the past. The use of a systematic approach such as has been utilized in this study is recommended, and the consideration of the recommendations made here.

There is a small but growing body of literature on HSPA in LMICs. However the bulk of the research and documented experiences on HSPA is from HICs. There is need for more research on HSPA to build a robust body of literature in LMICs. Some of the areas that have been identified for further research by this study, focusing on sub-national HSPA include: further exploration of alternative approaches like hierarchical cluster analysis for the analysis and presentation of data to support sub-national decision making; exploring the utilization of HSPA for downward accountability to communities in the context of decentralization and multiparty democracy in LMICs; and consideration of SDH in HSPA.

## Abbreviations

ACAD: Academia
AHSPR: Annual Health Sector Performance Report
AIDS: Acquired Immune Deficiency Syndrome;
ART: Anti-retrovial therapy
DHO: District Health Officer
DLT: District League Table
DPT: Diptheria Pertussis Tetanus
DTECH: District Technical Manager
GHIs: Global Health Initiatives
GNI: Gross National Income
HCA: Hierarchical Cluster Analysis
HCT: HIV Counselling and Testing
HICs: High Income Countries
HIV: Human Immune-deficiency Virus
HMIS: Health Management Information System
HSD: Health sub district
HSPA: Health System Performance Assessment
HSSP: Health Sector Strategic Plan
IA: International Agency
IPT: Intermittent Presumptive Treatment (of malaria in pregnancy)
LICs: Low Income Countries
LMICs: Low and Middle Income Countries
MDGs: Millennium Development Goals
MoH: Ministry of Health
NGO: Non- Governmental Organisation
NHP: National Health Policy;
NMS: National Medical Stores
OPD: Outpatient Department
OPM: Office of the Prime Minister
PHC: Primary Health Care
PMTCT: Prevention of mother to child transmission (of HIV/AIDS)
PNFP: Private not for Profit
QIIs: Quality Improvement Initiatives
SWAp: Sector Wide Approach to health development
TB: Tuberculosis
